# Sex and Estrous Cycle Effects on Anxiety- and Depression-Related Phenotypes in a Two-Hit Developmental Stress Model

**DOI:** 10.3389/fnmol.2019.00074

**Published:** 2019-04-11

**Authors:** Ivana Jaric, Devin Rocks, Heining Cham, Alice Herchek, Marija Kundakovic

**Affiliations:** ^1^Department of Biological Sciences, Fordham University, Bronx, NY, United States; ^2^Department of Psychology, Fordham University, Bronx, NY, United States

**Keywords:** early life stress, adolescence, sex difference, anxiety, depression, estrogen, epigenetic, gene expression

## Abstract

Stress during sensitive developmental periods can adversely affect physical and psychological development and contribute to later-life mental disorders. In particular, adverse experiences during childhood dramatically increase the risk for the development of depression and anxiety disorders. Although women of reproductive age are twice as likely to develop anxiety and depression than men of the corresponding age, little is known about sex-specific factors that promote or protect against the development of psychopathology. To examine potential developmental mechanisms driving sex disparity in risk for anxiety and depression, we established a two-hit developmental stress model including maternal separation in early life followed by social isolation in adolescence. Our study shows complex interactions between early-life and adolescent stress, between stress and sex, and between stress and female estrogen status in shaping behavioral phenotypes of adult animals. In general, increased locomotor activity and body weight reduction were the only two phenotypes where two stressors showed synergistic activity. In terms of anxiety- and depression-related phenotypes, single exposure to early-life stress had the most significant impact and was female-specific. We show that early-life stress disrupts the protective role of estrogen in females, and promotes female vulnerability to anxiety- and depression-related phenotypes associated with the low-estrogenic state. We found plausible transcriptional and epigenetic alterations in psychiatric risk genes, *Nr3c1* and *Cacna1c*, that likely contributed to the stress-induced behavioral effects. In addition, two general transcriptional regulators, Egr1 and Dnmt1, were found to be dysregulated in maternally-separated females and in animals exposed to both stressors, respectively, providing insights into possible transcriptional mechanisms that underlie behavioral phenotypes. Our findings provide a novel insight into developmental risk factors and biological mechanisms driving sex differences in depression and anxiety disorders, facilitating the search for more effective, sex-specific treatments for these disorders.

## Introduction

Stressful experiences during sensitive developmental periods can have a major impact on later-life physical and mental health (Romeo and McEwen, 2006; McEwen, 2008). In particular, adverse experiences during childhood, such as abuse or neglect, dramatically increase the risk for the development of psychiatric disorders including depression, anxiety disorders, and substance abuse (Heim and Nemeroff, 2001; Lupien et al., 2009). It has been proposed that stress in early life induces increased reactivity within the hypothalamic-pituitary-adrenal (HPA) axis, sensitizing an individual to future stressful situations and challenges that may precipitate psychiatric disorders (Lupien et al., 2009). It is notable, though, that only a subset of individuals that experience early-life trauma develop a psychiatric condition in their adult life, and it is of great interest to reveal the factors that promote both risk and resilience to the effects of early life adversity.

The majority of psychiatric conditions linked to early-life adversity show a substantial sex bias in prevalence and severity, yet little is known about sex-specific factors that promote or protect against the development of psychopathology (Altemus et al., 2014). One of the major reasons for the dearth of knowledge in this area has been a traditional focus on male subjects in experimental neuroscience research (Beery and Zucker, 2011; Will et al., 2017). Some insights have been provided by the studies of depression and anxiety disorders, which show striking sex bias being twice as frequent in women than in men (Kuehner, 2017; Li and Graham, 2017). The increased risk in females emerges with the onset of puberty and various findings have suggested that natural hormonal shifts predispose females to stress-related, mood and anxiety disorders (Steiner et al., 2003; Altemus et al., 2014; Kuehner, 2017; Li and Graham, 2017). How sex hormones and stress may interact to bring about an increased vulnerability in females remains unknown.

To examine potential developmental mechanisms driving sex disparity in risk for anxiety and depression, we established a two-hit developmental stress model including maternal separation in early life followed by social isolation in adolescence. Repeated and prolonged separations of offspring from their mothers is one of the most frequently used rodent paradigms modeling aspects of childhood maltreatment in humans (Heim and Nemeroff, 2001; Kundakovic and Champagne, 2015). This model has been associated with disrupted brain development, and emotional and cognitive dysregulation in later life, showing translational validity (Heim and Nemeroff, 2001; Kundakovic and Champagne, 2015). However, the results have been inconsistent among different laboratories, largely due to different rodent strains used as well as different nature and timing of manipulation (Murthy and Gould, 2018). We use an established maternal separation protocol in C57BL/6 mice, combining maternal deprivation with disrupted maternal care, which was previously shown to induce depression-like behavior in early adolescent male and female animals (Kundakovic et al., 2013b). The rationale of the current study was to introduce another stress hit in adolescence and to examine whether increased female vulnerability to stress may emerge as females start experiencing cycling sex hormone levels following the onset of puberty. We chose a “suboptimal,” mild adolescent stress paradigm, 3-week social isolation, previously shown to be insufficient to trigger behavioral abnormalities but exerting a synergistic disruptive effect on depression-related behavior when combined with another psychiatric risk factor (Niwa et al., 2013).

Our study provides a comprehensive analysis of the effects of early-life stress and adolescent stress, individually and in combination, on anxiety- and depression-related behavioral phenotypes in young adult male and female mice, accounting for the effects of the naturally cycling estrogen levels in females. Using several candidate genes of relevance to these behaviors, we also attempted to find molecular correlates that would link the effect of stressors to behaviors of interest. We examined both gene expression and DNA methylation in the ventral hippocampus, an area essential for emotional regulation, looking for a possible epigenetic link between stressful exposures and behavioral outcomes. We find complex interactions between two stressors, sex, and female estrogen status, and provide a new biological insight into the increased female vulnerability to stress and stress-related disorders.

## Materials and Methods

**Figure 1:**
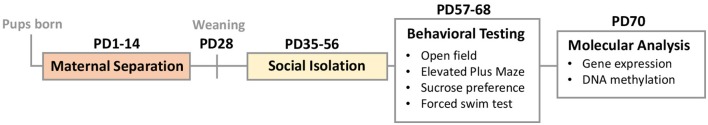
Study design: two stress hits included early-life maternal separation (MS, PD1–14) and adolescent social isolation (SI, PD35–56). This design generated four groups of animals: no stress controls (CON), MS only, SI only, and combined two-hit stress MS_SI group. The majority of animals underwent behavioral testing from PD57–68 and their non-tested littermates were sacrificed for molecular analyses of the ventral hippocampus at PD70.

Study Design (Figure 1): to assess the effects of early-life stress and adolescent stress, individually and in combination, on behavioral and molecular outcomes, the following treatment groups were generated: (1) no stress (CON); (2) early life stress—maternal separation from postnatal day (PD) 1–14 (MS); (3) social isolation in adolescence from PD35–56 (SI); and (4) two-hit stress group—early life MS + SI in adolescence (MS_SI). In all groups, behavioral testing was performed from PD57–68, during the animals’ light cycle, and in the following order (from least to most stressful): open-field test; elevated plus maze; sucrose preference; and forced swim test. Animals were sacrificed 2 days after the last behavioral test (PD70). Molecular analyses were performed on littermates that were not behaviorally tested to avoid the effect of behavioral testing on gene expression and DNA methylation. To avoid possible litter effects, a maximum of two pups per sex per litter were tested on behavioral measures, and one pup per sex per litter was used for molecular analyses.

### Animals

Male (*N* = 40) and female (*N* = 80) C57BL/6 mice were ordered from Jackson Laboratories and housed in same-sex groups of five for 2.5 weeks prior to mating to habituate to the facility. Mating pairs consisting of two females and one male were separately housed and yielded *N* = 72 litters which were counted and weighed at PD0. Eighteen litters were randomly assigned to each group (CON, MS, SI, and MS_SI). Litters were weighed again on PD14, and individuals from the litters were weighed on weaning day, PD28, at which point they were housed in same-sex and same-group cages of five. Regardless of the treatment group, all animals were group-housed until PD35. Animals were kept on a 12:12 light/dark cycle (lights on at 8 a.m.) and given *ad libitum* access to food and water. This study was carried out in accordance with the recommendations of the Institutional Animal Care and Use Committee at Fordham University. The protocol was approved by the Institutional Animal Care and Use Committee at Fordham University.

### Early-Life Stress

We used a previously established maternal separation protocol in C57BL/6 mice (Kundakovic et al., 2013b). Litters selected for early-life stress (MS and MS_SI groups) underwent maternal separation for 3 h every day for 2 weeks (PD1–14). During this time the dam was removed and placed in a temporary housing cage with food and water available. The time of day that maternal separation occurred was changed daily so that the stress was unpredictable. At a random time within the 3-h maternal separation window, dams were exposed to either restraint stress or forced swim to reduce the possibility of the dams giving compensatory maternal care to their litters following separation which was previously reported (Millstein and Holmes, 2007). In fact, we observed that this unpredictable stress disrupts maternal behavior for at least an hour after dams return to their pups; MS mothers spend more time out of nest and show reduced nurturing behaviors including less nursing, arched-back nursing, and licking/grooming, compared to control mothers (Supplementary Figure S1). Restraint stress lasted 20 min and involved placing the dam in a conical tube that restricted movement. Forced swim lasted 2 min and involved placing the dam in a 2 L glass beaker with 1 L of water, after which the dams were dried and returned to the temporary housing cage.

### Adolescent Stress

Animals selected for adolescent stress (SI and MS_SI groups) underwent social isolation for 3 weeks starting at PD35 and ending at PD56. Social isolation involved placing individual animals in separate cages without cagemates. Socially isolated animals were given *ad libitum* access to food and water. At PD56, all animals were again housed in same-sex, same-treatment groups.

### Open-Field Test

On PD57, animals from each group were tested using the Open Field paradigm. At the time of testing the animal was placed in the corner of the 40 cm × 40 cm × 35 cm glass box apparatus (Stoelting). The movement was tracked for 15 min using a camera and ANY-maze video tracking software (ANY-maze 5.1, Stoelting Co., Wood Dale, IL, USA). The size of the center zone in the open-field test was 20 cm × 20 cm. The total distance traveled, the amount of time the animal spent in the center of the maze vs. the periphery, and the latency to the first center entry was recorded. The results of the first 2 min of the test were used for the analysis.

### Elevated Plus Maze Test

On PD60, animals from each group were tested using the Elevated Plus Maze paradigm. At the time of testing the animal was placed in the center of the apparatus, which was a raised (50 cm) plus-shaped platform with two 35 cm × 5 cm arms, one being protected by 15 cm walls (Stoelting). The movement was tracked for 5 min using a camera and ANY-Maze video tracking software. The total distance traveled, the amount of time the animal spent in the open arms of the maze vs. the closed arms, and the latency to the first open arm entry were recorded. The results of the first 2 min of the test was used for the analysis.

### Sucrose Preference Test

From PD62–65, animals from each group were individually housed and given access to two water bottles with one containing 1% sucrose solution. Water bottles were weighed daily to assess sucrose preference. The position of the water bottles was switched each day to eliminate the possibility of attaining erroneous results due to place preference. The percentage of the sucrose solution consumed over the total volume of fluid consumed was reported as sucrose preference.

### Forced Swim Test

On PD68, animals from each group were tested using the Forced Swim paradigm. Animals were placed in 2 L glass beakers filled with 1 L of water. The movement was tracked for 6 min using a camera and ANY-Maze video tracking software. The amount of time the animal spent frozen (immobile) and the latency to freeze were recorded. To ensure the accuracy of data obtained by automated video tracking of this test, the data were additionally evaluated and confirmed by watching the video recordings.

### Estrous Cycle Stage Identification

To account for the sex hormone status of female animals, vaginal smears were taken immediately after behavioral testing or after sacrificing (for molecular analyses) for all female animals. Smears were analyzed by cytology and estrous cycle stage was recorded following previously established protocols (McLean et al., 2012). Briefly, smears were allowed to dry and then stained with crystal violet dye. Smears were then analyzed using light microscopy. Proestrus is characterized by a high number of nucleated epithelial cells. Estrus is characterized by a high number of cornified epithelial cells. Metestrus is characterized by mostly cornified epithelial cells, some nucleated epithelial cells, and some leukocytes. Diestrus is characterized by cornified epithelial cells, nucleated epithelial cells, and many more leukocytes compared to metestrus. For the purpose of behavioral data analysis (for each test except for the sucrose preference test), female animals were classified into two groups based on the estrogen status on the day of test: (1) *high-estrogen status* (proestrus, diestrus/proestrus transition, and proestrus/estrus transition); and (2) *low-estrogen status* (diestrus, estrus, metestrus, estrus/metestrus transition, and metestrus/diestrus transition).

### RNA and DNA Isolation

From each group, a subset of male (*n* = 8/group) and female (*n* = 12/group) animals which did not undergo behavioral testing were sacrificed at PD70 for molecular analysis. We checked the estrous cycle stage at the time of sacrificing, and as much as possible, we balanced the number of high- and low-estrogenic females in each group. Immediately after sacrifice, whole brains were isolated, frozen, and then stored at −80°C. The ventral hippocampus was later dissected, the tissue was immediately homogenized and DNA and RNA were extracted simultaneously using the Qiagen AllPrep DNA/RNA Mini Kit following the manufacturer’s instructions. This served as the starting material for all subsequent molecular analyses.

### Gene Expression Analysis

The analysis of gene expression was performed as described previously (Kundakovic et al., 2013a). Briefly, cDNA was synthesized from 500 ng of total RNA using the SuperScript III First-Strand Synthesis kit (Invitrogen, Carlsbad, CA, USA) following the manufacturer’s protocol. qRT-PCR was then performed in QuantStudio 3 (Applied Biosystems, Foster City, CA, USA) using Fast SYBR Green Master Mix (Applied Biosystems, Foster City, CA, USA) and RT-PCR primers specific to the targeted genes (Supplementary Table S1). Relative mRNA expression was calculated using the standard ΔΔCT method. *Ppia* gene was used as a reference gene, previously shown not to be affected by early-life stressors (Kundakovic et al., 2013a, b). All data are expressed relative to the male control group.

### DNA Methylation Analysis

DNA methylation analysis was performed at single-base resolution using the bisulfite pyrosequencing method as previously described (Kundakovic et al., 2013a). Briefly, 250 ng of genomic DNA was used for bisulfite conversion (EpiTect Bisulfite Kit, Qiagen) and 20 ng of bisulfite converted DNA was run in a PCR using the PyroMark PCR kit (Qiagen) and primers for genes of interest, one of which was biotinylated at the 5′ end. Biotinylated PCR products were processed on a vacuum workstation and pyrosequencing was performed on an Advanced PyroMark Q24 pyrosequencer using the advanced CpG reagents (Qiagen) and a specific sequencing primer. PCR and pyrosequencing primers for the mouse *Nr3c1* and *Cacna1c* genes are presented in Supplementary Table S2. Methylation levels of single CpG sites were quantified using PyroMark Q24 Advanced software (Qiagen).

### Statistical Analysis

For all analyses, we did two-way analysis of variance (ANOVA) with group and sex as factors. We first looked for the group by sex interaction, and if the interaction was not significant, it was dropped from the model and we tested the main effects of group and sex. For the analyses of behavior (with the exception of sucrose preference), we did an additional two-way ANOVA analysis in females in which we assessed the effects of group and estrogen status and their interaction. If the group by estrogen interaction was not significant, it was dropped from the model, and we assessed the main effects of group and estrogen status. All *post hoc* tests were performed using Tukey’s method. The analyses were performed in R version 3.5.1 and the graphs were generated using the R ggpubr package. Box plots show the first and third quartiles; the horizontal line is the median; the added, inner circles mark the mean with standard error for each box and small, outer circles mark possible outliers. The results were considered significant at a significance level of *P* < 0.05.

## Results

### Body Weight

**Figure 2:**
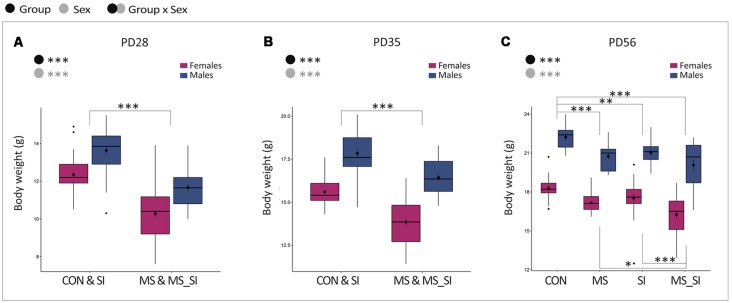
The effect of maternal separation and social isolation on body weight. Body weight was examined after weaning **(A)**, prior to social isolation **(B)**, and before behavioral testing **(C)**. Note that PD28 and PD35 show the effects of MS only and the groups are merged to represent animals that did not undergo MS (CON and SI groups) vs. animals that underwent MS (MS and MS_SI groups). At PD56, social isolation was completed and four groups of animals are shown separately reflecting the effects of both MS and SI hits (*N* = 15–23/sex/group). Symbols in the upper left corner of each graph show significant main effects of group and sex or their interaction. Significant results of *post hoc* tests are presented in detail. CON, control; MS, maternal separation; SI, social isolation; MS_SI, combined maternal separation and social isolation. *P < 0.05; **P < 0.01; ***P < 0.001.

We monitored the effect of stressors on body weight throughout the study, including post-weaning (PD28), prior to social isolation (PD35), and after social isolation (PD56) time points (Figure 2). At all time points, males had higher body weight compared to females, and this relationship was not affected by any of the treatments (no significant group by sex interaction was found). Maternal separation led to significantly reduced body weight in both male and female offspring which was evident at PD28 (*P* < 0.001, Figure 2A) and persisted through PD35 (*P* < 0.001, Figure 2B) and PD56 (*P* < 0.001, Figure 2C). At PD56, we found that social isolation in males and females also led to a significant reduction in body weight compared to control mice (*P* = 0.002, Figure 2C). We found no significant difference between the effects of maternal separation and social isolation on body weight (*P* = 0.6, Figure 2C). However, the body weight reduction was most profound in mice that underwent the double-stress paradigm, surpassing the effects of a single exposure to either maternal separation (*P* = 0.02) or social isolation (*P* < 0.001, Figure 2C).

### Anxiety-Like Behavior

In all four groups of animals, we performed two well-established tests for anxiety-like behavior: open-field and elevated plus maze tests.

#### Open Field

**Figure 3:**
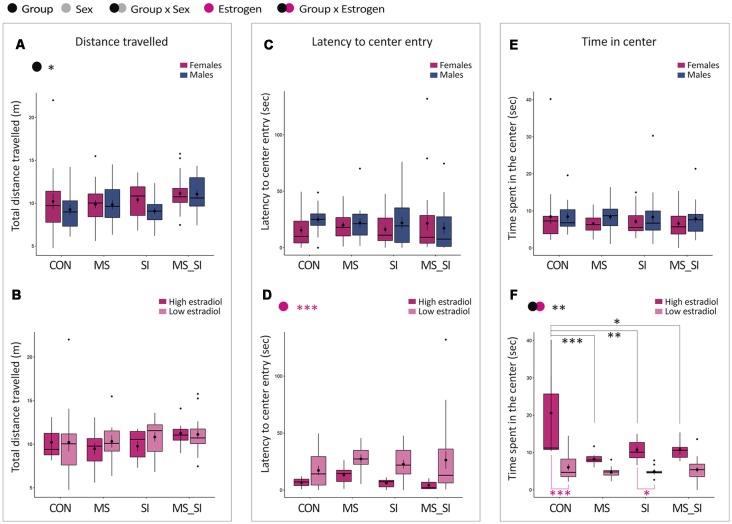
Sex-specific and estrous cycle-dependent effects of maternal separation and social isolation on the open field behavior. Total distance traveled **(A,B)**, latency to the first center entry **(C,D)** and time spent in the center **(E,F)** were analyzed, either in all animals with group and sex as factors (upper panels—**A,C,E**) or in females with group and estrogen status as factors (lower panels—**B,D,F**); (*N* = 15–23/sex/group). Symbols in the upper left corner of each graph show significant main effects of group and sex/estrogen or their interaction. Significant results of *post hoc* tests are presented in detail. CON, control; MS, maternal separation; SI, social isolation; MS_SI, combined maternal separation and social isolation. *P < 0.05; **P < 0.01; ***P < 0.001.

In the open field, we found no significant group by sex interaction on total distance traveled; however, we found a significant effect of group on this variable (*F*_(3,148)_ = 2.87, *P* = 0.04; Figure 3A). *Post hoc* test revealed that this effect was driven by the double-stress (MS_SI) group which was marginally more active than any other group including CON (*P* = 0.08), MS (*P* = 0.09), and SI (*P* = 0.08). The average increase in the activity was higher in male MS_SI (11.07 ± 0.52 m) vs. CON (9.27 ± 0.60 m) comparison than in female MS_SI (11.15 ± 0.41 m) vs. CON (10.22 ± 0.90 m) comparison. There was no difference in activity between CON, MS, and SI groups (*P* > 0.99 for all comparisons). No significant effect of group or estrogen, or an interaction between group and estrogen were found when females were considered separately from males (Figure 3B).

When we analyzed the latency to the first entry into the center of the open field, no significant effect of group or sex nor their interaction was found with regard to this variable (Figure 3C). However, we found a significant effect of estrogen status (*F*_(1,82)_ = 13.5, *P* < 0.001); high-estrogenic females showed shorter latency time to enter the center compared to low estrogenic females (Figure 3D).

Finally, we analyzed the time spent in the center of the open field. We found no significant group by sex interaction and no significant main effects of group or sex on this variable (Figure 3E). However, when females were considered separately, we found a significant group by estrogen interaction (*F*_(3,79)_ = 5.47, *P* = 0.002; Figure 3F). In addition, both main effects of group (*F*_(3,79)_ = 9.12, *P* < 0.001) and estrogen status (*F*_(1,79)_ = 39.34, *P* < 0.001) were significant. In general, high estrogenic females showed more time spent in the center of the open field than low estrogenic females. Interestingly, in *post hoc* test, the effect of group was found to be significant in high-estrogenic females only; MS (*P* < 0.001), SI (*P* = 0.003), and MS_SI (*P* = 0.01) high estrogenic females were all found to spend significantly less time in the center of the open field than control high-estrogenic females. Another interesting observation is that a significant difference in the time spent in the center between high and low-estrogenic females was found only in control (*P* < 0.001) and SI (*P* = 0.01) groups, whereas this difference was lost in groups that were exposed to early life stress: MS (*P* = 0.32) and MS_SI (*P* = 0.09) groups (Figure 3F).

#### Elevated Plus Maze

**Figure 4:**
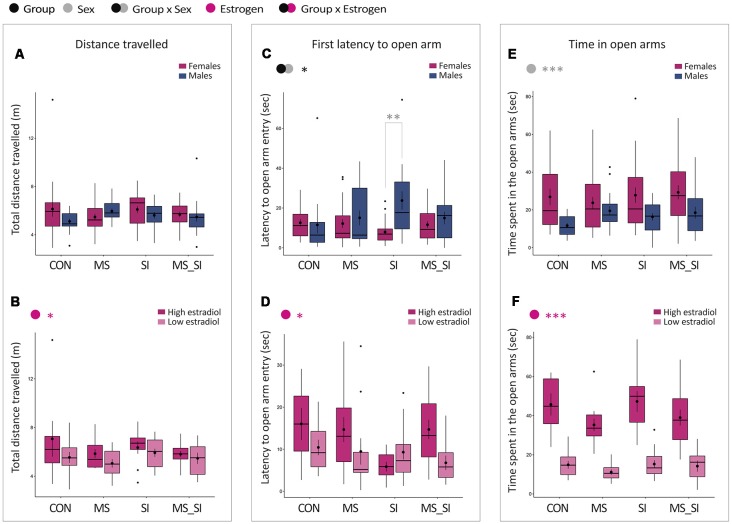
Sex-specific effects of maternal separation and social isolation in the elevated plus maze test. Total distance traveled **(A,B)**, latency to the first open arm entry **(C,D)** and time spent in the open arms **(E,F)** were analyzed, either in all animals with group and sex as factors (upper panels—**A,C,E**) or in females with group and estrogen status as factors (lower panels—**B,D,F**); (*N* = 15–23/sex/group). Symbols in the upper left corner of each graph show significant main effects of group and sex/estrogen or their interaction. Significant results of *post hoc* tests are presented in detail. CON, control; MS, maternal separation; SI, social isolation; MS_SI, combined maternal separation and social isolation. *P < 0.05; **P < 0.01; ***P < 0.001.

No significant effect of group or sex nor their interaction was found on total distance traveled in the elevated plus maze (Figure 4A). There was a significant effect of estrogen status on activity in the maze (*F*_(1,82)_ = 4.28, *P* = 0.04) with high-estrogenic females showing marginally higher activity than low-estrogenic females (Figure 4B).

With regard to the latency to the first open arm entry, we found a significant group by sex interaction (*F*_(3,144)_ = 3.35, *P* = 0.02; Figure 4C). The effect of the group showed a trend toward significance in males only (*P* = 0.07) due to a longer latency to enter the open arm in SI male group compared to CON males (*P* = 0.1). Furthermore, the significant group by sex interaction that we observed was due to a sex difference induced in SI group, in which SI males showed significantly delayed latency to enter the open arms compared to SI female group (*P* = 0.002). No significant sex difference was found in CON, MS, or MS_SI groups (*P* > 0.99 for all comparisons). In addition, we found a significant effect of estrogen status in females (*F*_(1,82)_ = 4.39, *P* = 0.04); on average, high estrogenic females took longer to enter the open arm than low estrogen females (Figure 4D).

We found no significant group by sex interaction and no significant effect of group on time spent in the open arms of the elevated plus maze (Figure 4E). However, we found a significant effect of sex (*F*_(1,148)_ = 17.78, *P* < 0.001), with females spending, on average, more time in the center of the maze than males (Figure 4E). This effect appears to be primarily driven by the estrogen status which was found to have a significant effect on time spent in the open arms in females (*F*_(1,82)_ = 131.85, *P* < 0.001), with high estrogenic females spending on average much longer time in the open arms than low estrogenic females (Figure 4F).

### Depression-Like Behavior

Two well-established tests for depression-like behavior were performed in all four groups of animals: sucrose preference and forced swim test.

#### Sucrose Preference Test

**Figure 5:**
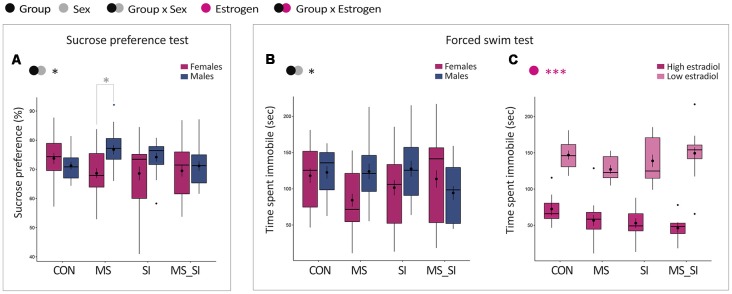
Sex-specific effects of maternal separation and social isolation on depression-related phenotypes. Results of sucrose preference **(A)** and the forced swim **(B,C)** tests are shown. Sucrose preference was analyzed in all animals with group and sex as factors **(A)**. For the forced swim test, time spent immobile was analyzed in all animals with group and sex as factors **(B)** and in females with group and estrogen status as factors **(C)**; (*N* = 15–23/sex/group). Symbols in the upper left corner of each graph show significant main effects of group and sex/estrogen or their interaction. Significant results of *post hoc* tests are presented in detail. CON, control; MS, maternal separation; SI, social isolation; MS_SI, combined maternal separation and social isolation. *P < 0.05; ***P < 0.001.

This test represents a model of anhedonia in humans and we found a statistically significant interaction between the effects of group and sex on sucrose preference in our study (*F*_(3,145)_ = 2.87, *P* = 0.04; Figure 5A). This interaction was primarily driven by a sex difference in sucrose consumption observed in MS group; MS females showed a significantly decreased sucrose preference when compared to MS males (*P* = 0.046). Sex difference in sucrose preference did not exist in control group (*P* = 0.99) or in other treatment groups, SI (*P* = 0.39) and MS-SI (*P* > 0.99) groups. Of note, we did not examine the effect of estrogen status in this test because the test spans 3 days or more than a half of the mouse estrous cycle.

#### Forced Swim Test

We analyzed time spent immobile in the forced swim test and found a significant group by sex interaction in this test (*F*_(3,145)_ = 3.07, *P* = 0.03; Figure 5B). In *post hoc* tests, we found no significant difference between groups and only trend for an immobility time difference between males and females in the MS group (*P* = 0.13). In females, we found no significant group by estrogen interaction and no significant main effect of group. However, we found a significant effect of the estrogen status (*F*_(1,82)_ = 211.80, *P* < 0.001), with high-estrogenic females generally having significantly shorter immobility time in the forced swim test than low estrogenic females (Figure 5C).

### Gene Expression Analysis

We determined the effects of early-life stress and adolescent stress on expression of the following candidate genes in the ventral hippocampus: *Bdnf* (encoding brain-derived neurotrophic factor); *Nr3c1* (encoding glucocorticoid receptor); *Egr1* (encoding early growth response protein 1)*; Cacna1c* (encoding calcium voltage-gated channel subunit alpha1C), *Gria3* (encoding glutamate ionotropic receptor AMPA type subunit 3); and *Dnmt1* (encoding DNA methyltransferase 1). The ventral hippocampus was selected because it is strongly implicated in emotion regulation and anxiety-related behavior (Bannerman et al., 2003; Fanselow and Dong, 2010; Kheirbek et al., 2013). The *Bdnf* and *Nr3c1* genes have been previously implicated in the effects of early-life stress and in psychopathology (Weaver et al., 2004; Angelucci et al., 2005; McGowan et al., 2009; Roth et al., 2009; Boulle et al., 2012; Kundakovic et al., 2013b, 2015). *Egr1* has been reported to be sensitive to stress and encodes an immediate early gene product, a transcription factor which regulates expression of numerous genes including *Nr3c1* (Weaver et al., 2004; Gutièrrez-Mecinas et al., 2011; Xie et al., 2013; Rusconi et al., 2016)*. Cacna1c* and *Gria3* are well-established psychiatric risk genes (Henley and Wilkinson, 2016; de Sousa et al., 2017; Dedic et al., 2018). Finally, *Dnmt1* encodes an epigenetic regulator that has been implicated in epigenetic effects of early-life stressors and in psychiatric disorders (Grayson and Guidotti, 2013; Kundakovic et al., 2013a; Dong et al., 2015).

**Figure 6:**
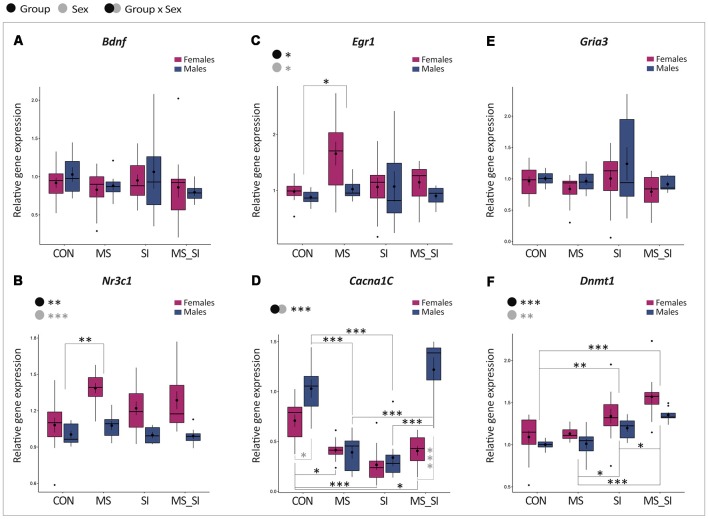
Sex-specific effects of maternal separation and social isolation on gene expression in the ventral hippocampus. The expression of *Bdnf* **(A)**, *Nr3c1* **(B)**, *Egr1* **(C)**, *Cacna1c* **(D)**, *Gria3* **(E)**, and *Dnmt1* **(F)** was analyzed in the ventral hippocampus, using group and sex as factors (*N* = 12 females/group, *N* = 8 males/group). Symbols in the upper left corner of each graph show significant main effects of group and sex or their interaction. Significant results of *post hoc* tests are presented in detail. CON, control; MS, maternal separation; SI, social isolation; MS_SI, combined maternal separation and social isolation. *P < 0.05; **P < 0.01; ***P < 0.001.

We found no significant group by sex interaction and no significant effect of group or sex on the expression of *Bdnf* in the ventral hippocampus (Figure 6A). With regards to *Nr3c1*, there was no significant group by sex interaction, but there were significant main effects of both group (*F*_(3,75)_ = 5.21, *P* = 0.003) and sex (*F*_(1,75)_ = 32.97, *P* < 0.001) on the expression of this gene (Figure 6B). *Post hoc* test showed a significant difference between MS and CON groups only (*P* = 0.001). This effect was driven by an increased *Nr3c1* expression in the MS female group (1.39 ± 0.04) compared to CON female (1.08 ± 0.06) and CON male (1.00 ± 0.03) groups, whereas the MS male group had *Nr3c1* expression (1.08 ± 0.04) comparable to controls (Figure 6B). In general, female mice also had a higher *Nr3c1* expression in the ventral hippocampus than males (*P* < 0.001; Figure 6B). *Egr1* showed a similar pattern of expression to *Nr3c1*; there was no significant group by sex interaction but there were significant main effects of group (*F*_(3,68)_ = 3.47, *P* = 0.02) and sex (*F*_(1,68)_ = 4.66, *P* = 0.03) on the expression of this gene (Figure 6C). The only two groups that showed a statistically significant difference in *Egr1* expression were MS and CON groups (*P* = 0.02) which, again, was driven by a higher expression in the MS female group (1.66 ± 0.21) compared to CON female (0.98 ± 0.06) and CON male (0.88 ± 0.05) groups and the MS male group had *Egr1* expression (1.03 ± 0.07) more comparable to controls (Figure 6C). In general, females had a higher *Egr1* expression compared to males (Figure 6C). The *Cacna1c* gene showed the most variable expression among the tested groups. We found a significant group by sex interaction for this gene (*F*_(3,72)_ = 14.75, *P* < 0.001; Figure 6D). In females, we found significantly reduced expression of this gene in all three treatment groups including MS (*P* = 0.02), SI (*P* < 0.001), and MS_SI (*P* = 0.02) compared to control female animals. In males, though, we found a significant reduction in *Cacna1c* expression in CON vs. MS comparison (*P* < 0.001) and in CON vs. SI comparison (*P* < 0.001) but there was no significant difference between CON and MS_SI groups (*P* = 0.63). However, the double-stress MS_SI male group had a significant higher expression than both single-stress exposed male groups—MS (*P* < 0.001) and SI (*P* < 0.001) groups. Of all groups, only CON (*P* = 0.03) and MS_SI groups (*P* < 0.001) showed a significant sex difference in *Cacna1c* expression; this difference was completely lost in MS and SI groups (*P* > 0.99 for both comparisons; Figure 6D). Unlike what we observed with *Cacna1c*, we found no significant group by sex interaction and no significant effect of group or sex on the expression of *Gria3* in the ventral hippocampus (Figure 6E). Finally, in terms of *Dnmt1* expression in the ventral hippocampus, there was no significant group by sex interaction but we found significant main effects of both group (*F*_(3,75)_ = 19.68, *P* < 0.001) and sex (*F*_(1,75)_ = 9.60, *P* = 0.003; Figure 6F). Two treatment groups SI (*P* = 0.003) and MS_SI (*P* < 0.001) showed a significant higher expression of *Dnmt1* than the control group; there was no significant difference between MS and control group (*P* = 0.97). Among the treatment groups, both SI (*P* = 0.01) and MS_SI groups (*P* < 0.001) had a significant higher expression of *Dnmt1* than MS group. The highest expression of *Dnmt1* was seen in the double-stress MS_SI group, significantly surpassing the effects of a single exposure to social isolation in the SI group (*P* = 0.01; Figure 6F).

### DNA Methylation Analysis

**Figure 7:**
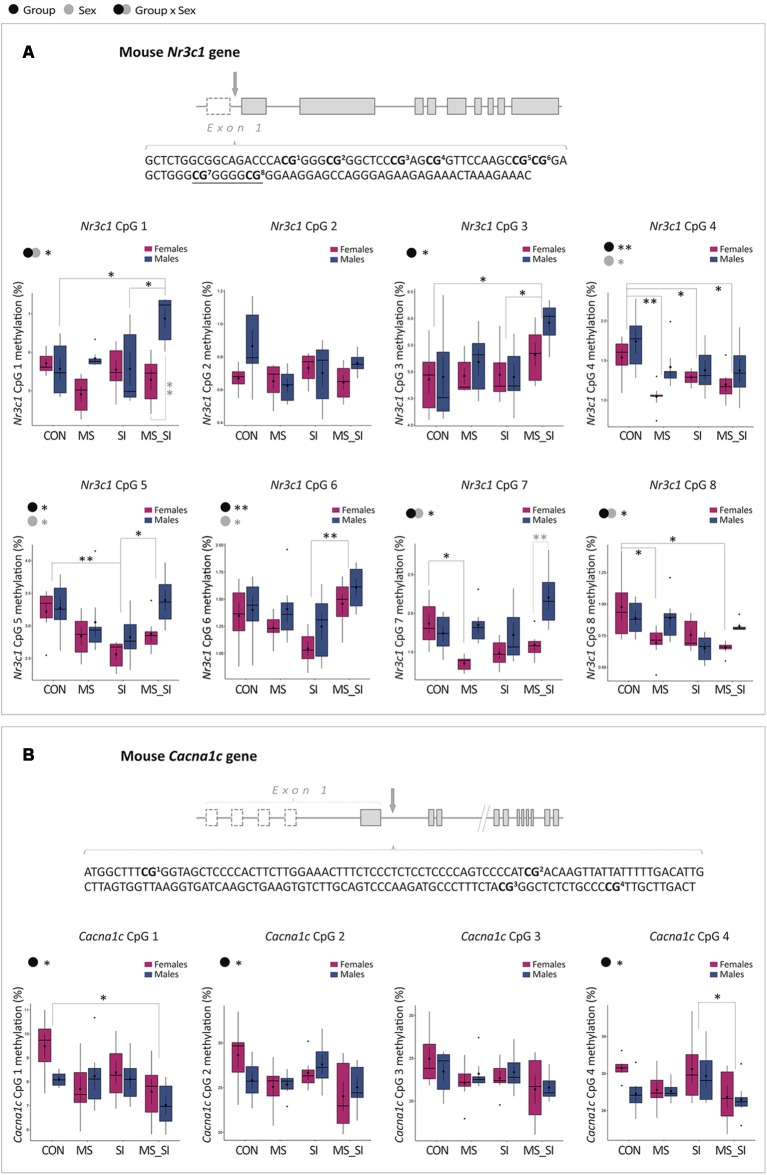
Sex-specific effects of maternal separation and social isolation on DNA methylation of the *Nr3c1* and *Cacna1c* regulatory regions. **(A)** Schematic representation of the *Nr3c1* gene, the sequence of the analyzed CpG sites in the *Nr3c1* 5’ regulatory region, and methylation levels of individual sites analyzed using group and sex as factors. Underlined sequence represents Egr1 binding site. **(B)** Schematic representation of the *Cacna1c* gene, the sequence of the analyzed CpG sites in the *Cacna1c* regulatory region, and methylation levels of individual sites analyzed using group and sex as factors (*N* = 6 sex/group; note: both genes have alternative first exons which are shown as dashed rectangles). Symbols in the upper left corner of each graph show significant main effects of group and sex or their interaction. Significant results of *post hoc* tests are presented in detail. CON, control; MS, maternal separation; SI, social isolation; MS_SI, combined maternal separation and social isolation. *P < 0.05; **P < 0.01.

To address possible epigenetic mechanisms underlying gene expression changes that we have observed, we performed DNA methylation analysis of the two candidate genes: *Nr3c1* and *Cacna1c*. We analyzed eight CpG sites located in the 5’ regulatory region of the *Nr3c1* gene, containing the binding site for transcription factor Egr1 (or NGFI-A) overlapping CpG sites 7 and 8 (Figure 7A; Weaver et al., 2004; Kundakovic et al., 2013b). For CpG1 site, we found a significant group by sex interaction (*F*_(3,40)_ = 4.67, *P* = 0.01; Figure 7A). Specifically, MS_SI males had significantly higher methylation at this site than CON (*P* = 0.02) and SI (*P* = 0.02) males. In addition, MS_SI males had higher methylation levels than MS_SI females (*P* = 0.003), whereas sex difference was not found in any other group. We found no significant main effects of group or sex nor their interaction when we analyzed CpG site 2 (Figure 7A). We found a significant main effect of group (*F*_(3,43)_ = 3.98, *P* = 0.01) on methylation levels of CpG site 3, which was driven by increased methylation in MS_SI group compared to CON (*P* = 0.02) and SI (*P* = 0.03) groups (Figure 7A). For CpG site 4, we found a significant effect of both group (*F*_(3,43)_ = 5.51, *P* = 0.003) and sex (*F*_(1,43)_ = 7.30, *P* = 0.01; Figure 7A). We found significantly lower CpG 4 methylation in MS (*P* = 0.003), SI (*P* = 0.04), and MS_SI (*P* = 0.01) groups compared to controls. In general, males had a higher methylation at CpG site 4 than females (*P* = 0.01). We found significant effect of both group (*F*_(3,43)_ = 4.91, *P* = 0.01) and sex (*F*_(1,43)_ = 5.77, *P* = 0.02) on CpG5 methylation (Figure 7A). This was driven by decreased methylation in the SI group compared to CON (*P* = 0.005) and MS_SI (*P* = 0.03) groups. In general, CpG 5 methylation was higher in males than in females. For CpG6, we found a significant effect of group (*F*_(3,43)_ = 4.95, *P* = 0.005) and sex (*F*_(1,43)_ = 4.12, *P* = 0.049; Figure 7A). The significant group difference was found in MS_SI to SI comparison only, where the SI group had significantly lower CpG6 methylation than MS_SI group (*P* = 0.002). Males had higher methylation levels than females. For CpG7 site, we found a significant group by sex interaction (*F*_(3,40)_ = 4.32, *P* = 0.01). A significant effect of group was found in MS vs. CON group comparison in females only (*P* = 0.04), where we found significantly reduced CpG7 methylation in MS females (Figure 7A). CpG7 methylation was also marginally higher in MS_SI male group when compared to CON male group (*P* = 0.09). These results were consistent with observed sex difference, which was statistically significant within MS_SI group only (*P* = 0.001) and showed a trend toward significance in MS group (*P* = 0.06), with CpG7 methylation being higher in males of both groups. Finally, we found a significant group by sex interaction with regards to methylation of CpG site 8 (*F*_(3,40)_ = 3.50, *P* = 0.02; Figure 7A). In *post hoc* test, we found only a significant decrease in CpG8 methylation in two female treatment groups—MS (*P* = 0.04) and MS_SI (*P* = 0.01)—compared to CON female group. No significant sex difference was observed in any of the groups.

We also analyzed four CpG sites in the first intron of the *Cacna1c* gene (Figure 7B). The analyzed region is within 1 kb from a transcription start site and is located in the CpG island shore having high homology between the mouse and human genome. In addition, these sites were previously shown to have cell-type specific methylation patterns (Nishioka et al., 2013). For CpG1, we found a significant main effect of group (*F*_(3,43)_ = 3.43, *P* = 0.03) which was driven by a decreased CpG1 methylation found in MS_SI group compared to CON group (*P* = 0.02; Figure 7B). For CpG2, we found a marginally significant effect of group (*F*_(3,43)_ = 2.79, *P* = 0.05) which was again driven by reduced CpG2 methylation in MS_SI group compared to CON group showing a trend toward significance (*P* = 0.1; Figure 7B). For CpG3, we found a similar trend with the main effect of group (*F*_(3,43)_ = 2.2, *P* = 0.1) due to a marginally significant reduction in CpG site 3 in MS_SI group compared to controls (*P* = 0.07; Figure 7B). There was also a significant effect of group on CpG4 methylation (*F*_(3,43)_ = 2.92, *P* = 0.04), again driven by reduced methylation in MS_SI group but this time compared to SI group (*P* = 0.04; Figure 7B). We found no significant effect of sex on methylation of any of the CpG sites analyzed in the *Cacna1c* gene.

## Discussion

In this study, we examined the effects of early-life stress and adolescent stress, individually and in combination, on body weight, anxiety- and depression-related behaviors, and expression and DNA methylation of genes implicated in stress-related psychiatric disorders. Overall, we found that a single, early-life stress exposure had the most profound effect on anxiety- and depression-related phenotypes in adult animals. Interestingly, the “second hit” of a suboptimal stress exposure in adolescence frequently attenuated the effects of early-life stress. Moreover, lasting effects of early-life stress are largely found to be female-specific and we have found significant effects of estrogen status on anxiety- and depression-like behavior in both control female animals and in those exposed to stress.

The current study is the continuation of our previous study which examined the effects of early-life maternal separation on early adolescent male and female offspring (Kundakovic et al., 2013b). The two developmental stressors that we used in the current study were sufficient to induce metabolic dysfunction in animals, as we have seen through the persistent reduction in body weight observed in all treatment groups. Interestingly, reduced body weight is a rare phenotype on which two stress hits worked synergistically, surpassing the effects of individual stress exposures. Another phenotype that emerged only in the double-hit stress group is hyperactivity in the open field test, with males being more strongly affected. While increased locomotor activity is a rather non-specific phenotype, which, interestingly, did not affect any of the indices of anxiety or depression-related behavior, this phenotype is seen after the treatment with psychostimulants and is reminiscent of hyperactivity seen in disorders such as attention deficit hyperactivity disorder (ADHD). Psychotic disorders and ADHD have been linked to developmental stress with males being more severely impacted (Bale et al., 2010), similar to what we observed in our study. Therefore, the double-stress paradigm that we constructed may be of relevance for studies of metabolic dysfunction and psychosis- or hyperactivity-related disorders but we found surprisingly small effects of this paradigm with regards to anxiety- and depression-related behaviors.

There are several possible explanations for the observed “buffering” effect of adolescent social isolation on the effect of early-life stress on anxiety- and depression-related phenotypes. First, 3 weeks of adolescent social isolation is a mild stressor. The only phenotype that we observed in the SI group is a marginally delayed latency to the open arm entry in males, which is as an index of increased anxiety-like behavior. Other than that, the behavioral effects of this paradigm were largely non-significant, in line with what was previously reported (Niwa et al., 2013). Our results suggest that mild stress in adolescence may decrease the risk for the development of anxiety and depression in individuals that experienced certain forms of early-life trauma. While this finding may be counterintuitive, it is consistent with the “mismatch hypothesis” of psychiatric disease (Schmidt, 2011; Nederhof, 2012). This hypothesis states that early-life environment induces adaptive phenotypic changes, preparing an individual for later-life environmental conditions (Nederhof, 2012). If the early-life and later-life environment match, the early-life stress may even be beneficial. However, the disorder may occur once the later-life conditions do not match the stressful environment experienced in early life. As an example, it was shown that rats who receive low levels of maternal care (form of early-life stress) exhibit enhanced learning under stressful conditions but impaired learning under normal conditions in later life (Champagne et al., 2008). There are also additional “two-hit stress” studies that showed increased stress resilience in animals that experienced early-life stress (Biggio et al., 2014; Hsiao et al., 2016; Santarelli et al., 2017). However, while the “mismatch hypothesis” is plausible to explain the lack of anxiety- and depression-related phenotypes in our two-hit stress model, it is likely that a stronger stressor during adolescence or adulthood would, indeed, worsen the phenotype induced by early-life stress, as seen in some studies (Peña et al., 2017). In addition, in humans, genetic vulnerability or resilience to depression and anxiety disorders, certainly play an important role in how early- and later-life stress affect the risk for the development of these disorders.

As compared to our previous study which showed that maternal separation induces decreased sucrose preference, a form of anhedonia or depression-like behavior, in both male and female early adolescent mice (Kundakovic et al., 2013b), here we show that this effect persists into adulthood only in female animals. It is well known that anxiety and depression show explicit sex bias with women of reproductive age being twice as likely as men of the corresponding age to develop these disorders. The mechanisms underlying this sex disparity are unknown, and the studies of early-life stress have not necessarily addressed this (Bale et al., 2010), particularly because a lot of studies were focused on male animals. Our study provides an important insight into this sex disparity, providing a clue about how early-life stress can be a sex-specific factor in driving vulnerability to anxiety and depression. Indeed, the early-life paradigm that we use, combining maternal separation with unpredictable maternal stress, turned out to be a good model to study increased female vulnerability to these disorders.

Among the most interesting sex-specific findings in our study is the effect of estrogen status and its interaction with early-life stress on anxiety- and depression-related behaviors in females. In fact, our most consistent finding is that the high-estrogen phase of the cycle is associated with remarkably lower indices of anxiety and depression-like behavior compared to the low-estrogen phase. This was basically seen in all tests in which estrogen status was controlled for, including: open field (shorter latency and longer time spent in the center in high-estrogenic females); elevated plus maze (longer time spent in the open arms in high estrogenic females); and forced swim test (shorter time spent immobile in high estrogenic females). Our results are consistent with earlier studies in rats (Marcondes et al., 2001; Walf and Frye, 2006) and clearly indicate the importance of estrogen status for anxiety and mood regulation in females of reproductive age. Indeed, we can conclude that high estrogen levels have a protective, anxiolytic effect whereas a physiological drop in estrogen may increase the risk for the occurrence of anxiety and depression symptoms in females of reproductive age. This is in line with human data which show that a large number of women with major depression experience worsening of their symptoms in the premenstrual, low-estrogen phase of the cycle, even when antidepressant therapy is effective during the remainder of the cycle (Kornstein et al., 2005; Altemus et al., 2014). Importantly, hormonal fluctuations, rather than low hormone levels *per se*, seem to be critical for both pathogenesis and treatment of depression, considering that post-menopausal women with stable low hormone levels appear to lose sensitivity to estrogen (Morrison et al., 2004) and show a similar risk for depression when compared to men of their age (Deecher et al., 2008; Altemus et al., 2014).

Our study provides an important, novel insight into how stress can interact with the ovarian cycle to facilitate the increased anxiety and depression vulnerability in females. When we focused our analysis on female animals, we found that developmental stressors had a significant negative impact on anxiety-like behavior (time spent in the center of the open-field) in our female population. However, when we looked further into the estrous cycle effects, we found that stressors increased anxiety-like behavior only during the high-estrogen phase of the cycle when the anxiety levels are typically low. No effect of stress was found during the low-estrogen phase when the anxiety levels are already high. In addition, the most significant effect was found in the early-life stress group. And, even more interestingly, maternally separated animals (MS and MS_SI groups) showed a largely blunted, insignificant difference in anxiety levels between low and high estrogen phases, suggesting that the anxiolytic effect of estrogen is predominantly lost in groups that experience early life stress. Whether this is the consequence of lower estrogen levels or lost sensitivity to estrogen in response to early-life adversity remains to be further explored. Nevertheless, this finding reveals a possible sex-specific mechanism through which early-life adversity may contribute to sex disparity in risk for anxiety and depression by suppressing the “protective” effect of estrogen in women of reproductive age.

We further explored gene expression in the ventral hippocampus with an intent to find molecular correlates of the behavioral effects that we have observed. For this analysis, we did not look at the estrous cycle effects but have explored more general sex-specific patterns of gene expression that may contribute to the sex difference in vulnerability. Interestingly, although changes in hippocampal *Bdnf* expression have been found in many studies of early-life adversity (Onishchenko et al., 2008; Roth et al., 2009; Kundakovic et al., 2013b, 2015; Boersma et al., 2014), we found no significant changes in the expression of this gene in our study. This may be due to the fact that we focused our analysis on the ventral hippocampus while previous studies examined the entire hippocampus. Considering that the ventral and dorsal hippocampi are functionally distinct regions (Fanselow and Dong, 2010), showing distinct gene expression profiles (Cembrowski et al., 2016) and distinct responses to chronic stress (Czéh et al., 2015; Pinto et al., 2015), changes previously reported may largely stem from the stressor-induced alterations in the dorsal hippocampus. However, in MS females, we found an overexpression of *Nr3c1*, which is another gene that has been repeatedly associated with early-life stress in both animals and humans (Weaver et al., 2004; Champagne et al., 2008; McGowan et al., 2009; Suderman et al., 2012; Kundakovic et al., 2013b). This gene encodes the glucocorticoid receptor, directly involved in the negative feedback regulation of the HPA axis, providing one of the possible mechanisms for HPA axis dysregulation and consequent behavioral changes in the MS female group. Another interesting finding is that *Egr1* shows an almost identical expression pattern as *Nr3c1*. Considering that the *Nr3c1* regulatory region contains the binding site for Egr1 (also known as NGFI-A; Weaver et al., 2004; Kundakovic et al., 2013b), and Egr1 is induced by stress (Xie et al., 2013; Rusconi et al., 2016), this provides a plausible link between coordinate up-regulation of these two genes and anxiety- and depression-related phenotypes in the early-life stress female group.

The *Cacna1c* gene was another important candidate gene in our study. This gene has recently received a lot of attention, as it is considered to be one of the strongest genetics hits in psychiatry, associated with risk for bipolar disorder, major depression, and schizophrenia (Ferreira et al., 2008; Sklar et al., 2008; Green et al., 2010). Moreover, embryonic deletion of this gene in the mouse forebrain results in increased anxiety-like behavior and increased susceptibility to chronic stress (Dedic et al., 2018). Some clinical and animal studies have also found a significant interaction between sex and *Cacna1c* genotype (Dao et al., 2010; Witt et al., 2014; Takeuchi et al., 2018); for instance, female, but not male, *Cacna1c* heterozygous mice have been shown to display decreased risk-taking behavior and increased anxiety in multiple tests (Dao et al., 2010). Our study has revealed that this gene is very sensitive to developmental stress and that, depending on the number of hits, the effects of stress can be sex-specific. First, we observed that each individual stressor, maternal separation and social isolation, decreased Cacna1c mRNA levels in both sexes. Once these two stressors are combined, though, females still show reduced expression, but this effect is lost in males. These results provide a good example where deficiency in a single gene cannot be simply associated with behavioral phenotype. For instance, we can speculate that the down-regulation of *Cacna1c* expression contributed to increased anxiety-like behavior observed in females of all groups and in SI males, with MS females being most significantly affected because these animals also show dysregulated expression of *Egr1* and *Nr3c1*. In addition, “normalized” *Cacna1c* expression is consistent with the lack of anxiety and depression-related phenotype in the double-stress male group. However, MS males also did not show any significant behavioral phenotype, despite reduced *Cacna1c* expression, suggesting that these animals may have expressed some protective or pro-resilient factors that “buffered” the effect of *Cacna1c* deficiency.

Finally, *Dnmt1* is the only gene which showed the largest expression changes in the double-hit stress group. Being an epigenetic regulator, Dnmt1 over-expression may affect the activity of many downstream target genes and, therefore, contribute to behavioral phenotypes. One possibility is that Dnmt1 up-regulation contributed to the hyperactivity seen in MS_SI group. This is consistent with the finding that the psychostimulant methamphetamine, which induces hyperactivity in rodents, also induces Dnmt1 mRNA levels in the rodent brain (Numachi et al., 2007). In addition, an increased *Dnmt1* expression has been found in psychosis (Veldic et al., 2005) and DNA methylation changes have been reported in many neuropsychiatric disorders including schizophrenia (Grayson and Guidotti, 2013) and ADHD (Heinrich et al., 2017; Chen et al., 2018), further providing a possible link between Dnmt1 dysregulation and the hyperactivity-related phenotype observed in our double-hit stress group.

In the last part of our study, we explored whether DNA methylation, an epigenetic mechanism, may contribute to the effects of developmental stressors on gene expression and behavioral outcomes. There are now a quite large number of studies suggesting that early-life experiences may exert long-term effects on brain and behavior *via* epigenetic mechanisms. Among those studies, changes in DNA methylation of the *Nr3c1* gene were the first to be linked to altered gene expression, HPA dysfunction, and anxiety-and depression-related phenotypes induced by early-life adversity in rats (Weaver et al., 2004) and humans (McGowan et al., 2009). Here we examined the region in mouse which is highly homologous (92%) to the previously reported exon 1_7_ in rats (Weaver et al., 2004), and also containing the binding site for Egr1 (or NGFI-A) transcription factor encompassing two CpG sites (Weaver et al., 2004; Kundakovic et al., 2013b). Changes in DNA methylation within or adjacent to the transcription factor binding sites are particularly poised to affect transcriptional activity by preventing or facilitating the binding of transcription factors (Klose and Bird, 2006). While our results show complex group- and sex-specific patterns, it is notable that both sites in the Egr1 binding motif show female-specific reduction in methylation in the MS group. This finding is consistent with higher *Nr3c1* and *Egr1* expression in this group, and provides one of the possible mechanisms for the induced expression of the glucocorticoid gene and consequent behavioral alterations in early-life stress female group.

In terms of *Cacna1c* gene, there are reports of altered or more variable DNA methylation patterns in bipolar disorder (Starnawska et al., 2016) and depression (Córdova-Palomera et al., 2015) patients, respectively, compared to healthy subjects. We analyzed the region close to the transcription start site, with high homology between mice and humans, and shown to be differentially methylated between neurons and glia cells (Nishioka et al., 2013), hence, of possible functional relevance. We found subtle changes in DNA methylation of this gene in the double-stress hit group only, which cannot explain group- and sex-specific differences in *Cacna1c* gene expression that we have observed. However, it is possible that other regions of the *Cacna1c* gene, not examined in this study, may show differential methylation, which remains to be further explored. In addition, we would like to note that, although our DNA methylation analysis was limited to two regions and only 12 CpG sites total, our results suggest that epigenetic dysregulation may be prominent in the double-stress group, in line with changed gene expression of an epigenetic regulator, Dnmt1.

## Conclusion

In conclusion, our study shows complex interactions between early-life and adolescent stress, between stress and sex, and between stress and female estrogen status in shaping behavioral phenotypes of adult animals. In our paradigm, early-life stress had the most significant impact on anxiety- and depression-related phenotypes and this was female-specific. We discovered that early-life stress disrupts the protective role of estrogen in females, and promotes female vulnerability to anxiety- and depression-related phenotypes associated with the low-estrogenic state. We found transcriptional and epigenetic alterations in psychiatric risk genes, *Nr3c1* and *Cacna1c*, that likely contributed to the stress-induced behavioral effects. In addition, two general transcriptional regulators, Egr1 and Dnmt1, were found to be dysregulated in maternally-separated females and in animals exposed to both stressors, respectively, providing insights into possible transcriptional mechanisms that underlie behavioral phenotypes. We envision future transcriptomics and epigenomics studies that will give more definitive answers regarding molecular signatures and mechanisms that drive developmental stress-induced vulnerability or resilience to anxiety and depression-related phenotypes. Our study highlights the importance of accounting for the effects of sex and female hormone status in the studies of the impact of stress on brain and behavior, and provides a foundation for the future studies of molecular mechanisms underlying sex disparity in anxiety and depression. This knowledge will be extremely important to enable the development of more effective, sex-specific treatments for these disorders.

## Author Contributions

IJ and MK designed the study. IJ, DR, AH, and MK performed the experiments. HC performed statistical analysis. IJ, DR, HC, and MK interpreted the data. IJ, HC, and MK constructed the figures. DR and MK wrote the article. MK conceived and directed the project. All authors revised and approved the final version of the article.

## Conflict of Interest Statement

The authors declare that the research was conducted in the absence of any commercial or financial relationships that could be construed as a potential conflict of interest.
