# Supplementary material for: Sex and Estrous Cycle Effects on Anxiety- and Depression-Related Phenotypes in a Two-Hit Developmental Stress Model
Source: Front Mol Neurosci. 2019 Apr 11;12:74. doi: 10.3389/fnmol.2019.00074 (PMC6470284; doi:10.3389/fnmol.2019.00074)
Supplement: Supplementary file 1 [file Data_Sheet_1.pdf]

Supplementary Figure 1

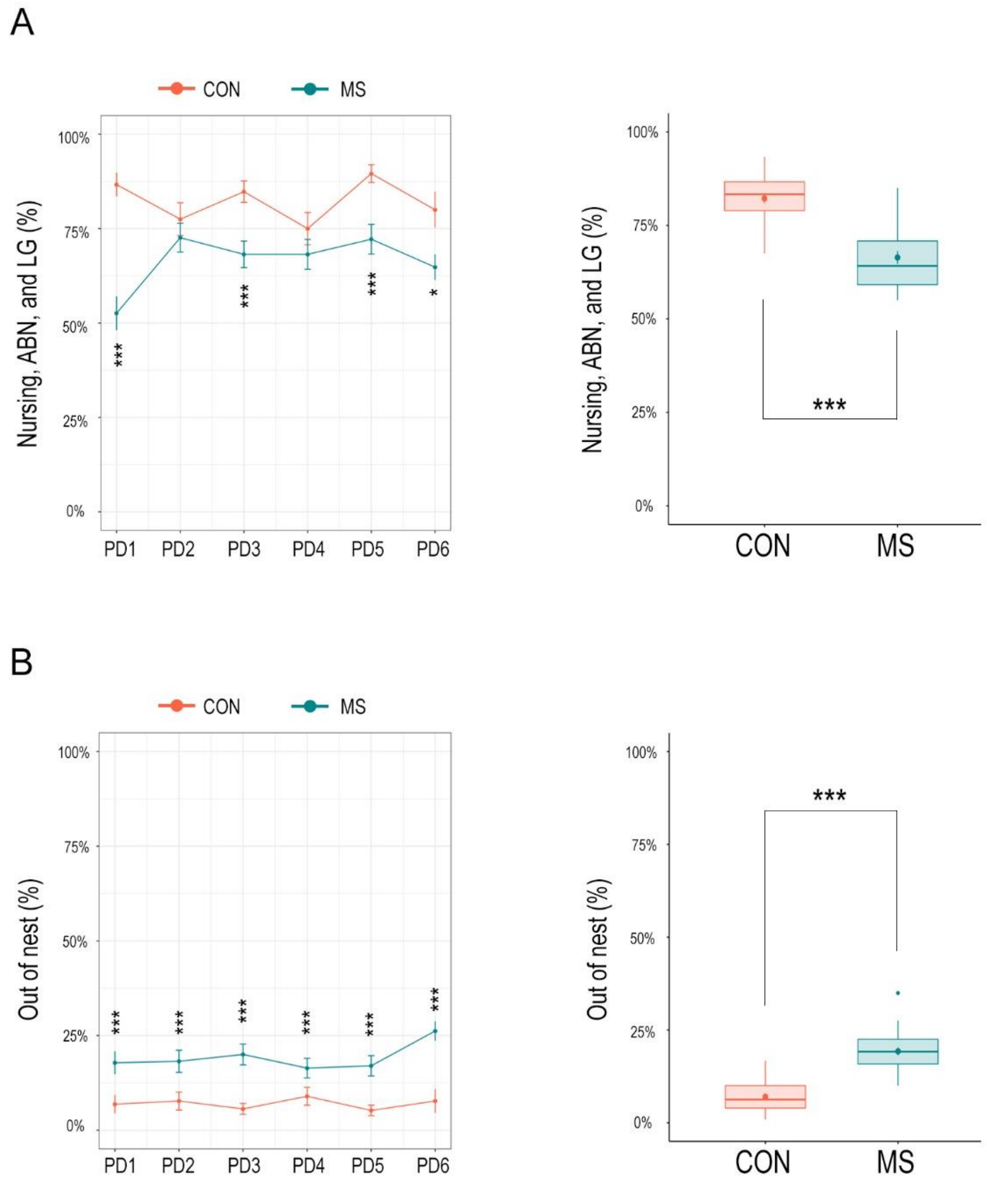

Maternal behavior in dams of control and MS groups. A. Frequency of nurturing behaviors in the first six days post-partum, including nursing, arched-back nursing (ABN), and licking-grooming (LG), is significantly reduced in the mothers of MS (maternal separation) group compared to CON (control) mothers, across days (as measured by two-way repeated measures ANOVA, with group and day as factors, left) and on average (as measured by t-test on averaged values for six days, right); **B.** MS dams spend significantly more time out of nest compared to control dams, across all days (left) and on average (right). (CON, N=24; MS, N=25); Maternal behavior was observed for an hour each day, following a three-hour mother-pup separation session; *, $P < 0.05$; ***, $P < 0.001$.

Supplementary Table 1. Gene expression analysis primers

Gene	Forward Primer	Reverse Primer
Bdnf	CATAAGGACGCGGACTTGTACA	AGACATGTTTGCGGCATCCA
Dnmt1	GCCATGTGAACAGGAAGATGAC	GTCCAAGTGAGTTTCCGGTCTT
Nr3c1	AACTGGAATAGGTGCCAAGC	GAGGAGAACTCACATCTGGT
Cacna1c	GGCTCTGCTGTGTCTGACC	TACTCCACTCGTTCCAGGTTGG
Egr1	AGCGAACAACCCTATGAGCAC	GGATAACTCGTCTCCACCATCG
Gria3	GGTCATTCTCACGGAGGATTCC	AGTGGTGTTCTGGTTGGTGTTG
Ppia	GAGCTGTTTGCAGACAAAGT	CCCTGGCACATGAATCCTGG

Supplementary Table 2. Primers for PCR and pyrosequencing

**Mouse *Nr3c1* gene**	
PCR primer - forward	GGTTTTGTAGGTTGGTTGTTATTT
PCR primer - reverse - Biotinylated	/5Biosg/TCTCTTCTCCCTAACTCCTT
Pyrosequencing primer	GGGTTTTGGAGGTAGATTTA
**Mouse *Cacna1c* gene**	
PCR primer - forward	/5Biosg/TGGTAAGGTAAGGAAAGATATAATGT
PCR primer - reverse - Biotinylated	ATCATCAACTCCCCTCTAATAATAAATA
Pyrosequencing primer	GTAAGGAAAGATATAATGTGG
